# Extirpation of the Uterus

**Published:** 1846-05

**Authors:** T. Gregson


*Extirpation of the Uterus.*—By T. Gregson, Esq.—Mrs. A——y had been delivered of her second child by a village surgeon two years ago. As she complained of much pain and uneasiness on the third day, her medical attendant ordered her to get out of bed and walk smartly across the floor. She recovered slowly and complained much. About a year ago she came here, and was some months under the care of a surgeon. About six months since I was called to attend her. I found her extremely emaciated and exsanguine, having, for above a year, been exhausted by most profuse hæmorrhage at every monthly period. On examination, I found a pear-shaped body filling the vagina, the os tincæ embracing it firmly, and apparently adhering on one side. I gradually introduced my fingers, endeavouring to grasp and push it through the os tincæ. This procedure caused extreme pain, and hæmorrhage, without the tumor yielding in the least. It was of a purplish red colour. Feeling satisfied that it was an almost complete eversion of the uterus, I endeavoured, by chalybeates, ergot, and astringents, to improve the system, but every monthly period produced extreme exhaustion, and death seemed inevitable. Being resolved, as a last chance, and herself and friends being willing, I determined to extirpate the uterus. I went, accompanied by my friend Mr. Frost, a most able accoucheur, who agreed with me that, as she was much exhausted and exsanguine, the attempt was justifiable. I laid hold of the tumor with the vulsellum, and drew it down as far as possible; in so doing the os tincæ entirely disappeared, leaving no doubt of the nature of the case. A very strong silk cord was then passed around it, and carried as high as possible by means of the double canula, the cord being also passed through the eye of a strong steel staff, very slightly curved in a bow-shape. I found this a very valuable means, as I could carry the ligature around the parts with the greatest facility. The knot was then tied with great firmness, leaving the staff included in the ligature, and opposite to the knot. This instrument was fastened to the thigh by a piece of tape. On the second day, by merely turning the handle of the staff once or twice, the ligature was greatly tightened; this was done from day to day, causing the rapid death of the part. It came away on the 9th day. During the time from the operation to its coming away, reaction was extremely moderate. She had an occasional anodyne, the catheter was used twice, and castor-oil was prescribed. She gained strength rapidly: she was made to keep the recumbent posture about twenty days. It is now three months since the operation was performed. She goes about her house, and has walked out a little, feeling easy and comfortable. These cases being generally considered hopeless, I have detailed matters perhaps minutely.

I believe the precautions taken, where the ligature is used to prevent inflammation, often produce the evils intended to be avoided; I allude to not tying the ligature too firmly at first. I think that no ligature can be tied too firmly, and that the great point to be aimed at is to destroy the vitality of the part at once, this being the surest means of preventing spreading inflammation and absorption of purulent or putrid matter.

On examining the part, I find that the entire body and neck of the uterus had been removed. There has not been the least disturbance at the monthly periods, nor symptoms of the system feeling the want of the organ removed.—*Medical Gazette.*

